# Feasibility of immunotherapy in cancer patients with persistent or past hepatitis B or C virus infection

**DOI:** 10.1002/jgh3.12737

**Published:** 2022-04-22

**Authors:** Tasuku Nakabori, Yutaro Abe, Sena Higashi, Takeru Hirao, Yasuharu Kawamoto, Shingo Maeda, Kazuma Daiku, Makiko Urabe, Yugo Kai, Ryoji Takada, Takuo Yamai, Kenji Ikezawa, Hiroyuki Uehara, Kazuyoshi Ohkawa

**Affiliations:** ^1^ Department of Hepatobiliary and Pancreatic Oncology Osaka International Cancer Institute Osaka Japan

**Keywords:** hepatitis B virus, hepatitis C virus, immunotherapy, liver injury

## Abstract

**Background and Aim:**

Immune checkpoint inhibitors (ICIs) can cause immune‐related adverse events in the liver. The risk of exacerbating liver injury is of concern in patients infected with hepatitis B virus (HBV) or hepatitis C virus (HCV), as immunotherapy can damage liver function because of the immune response against viral antigens. We assessed the feasibility of immunotherapy in HBV‐ or HCV‐infected patients.

**Methods:**

This retrospective study included 266 patients with persistent or past HBV infection, 26 patients seropositive for anti‐HCV, and 820 patients with negative viral markers for HBV and HCV, who were treated with ICIs. ICI‐induced liver injury and changes in virological markers were analyzed.

**Results:**

The occurrence rates of ICI‐induced liver injury in the HBsAg‐positive, anti‐HBc‐positive/anti‐HBs‐positive, and anti‐HBc‐positive/anti‐HBs‐negative groups were 12.5, 21.6, and 19.1%, respectively, which were comparable with those of the negative for HBV‐ and HCV‐related markers group (20.9%). The frequency of any grade ICI‐induced liver injury was different among the HCV RNA‐positive (3/5; 60.0%), anti‐HCV‐positive/HCV RNA‐negative (2/21; 9.5%), and negative for HBV‐ and HCV‐related markers (171/820; 20.9%) groups (*P* = 0.045), with no significant difference in grade ≥2 ICI‐induced liver injury. In patients with persistent infection, neither serum HBV DNA, HBsAg, nor HCV RNA level changed significantly during ICI treatment. One of five treatment‐naïve HCV‐infected patients required interruption of ICI treatment due to virus‐related liver injury.

**Conclusion:**

Immunotherapy is feasible for most cancer patients with chronic HBV or HCV infection; however, liver function and virological markers should be carefully monitored in treatment‐naïve patients, especially those with HCV infection, during ICI treatment.

## Graphical abstract

There were no significant differences in the frequency of any grade or grade ≥2 immune checkpoint inhibitor (ICI)‐induced liver injury in cancer patients with persistent or past hepatitis B virus (HBV) infection. No significant difference was observed in the frequency of grade ≥2 ICI‐induced liver injury in cancer patients positive for hepatitis C virus (HCV)‐related markers, though HCV‐RNA positive patients had a higher frequency of any grade ICI‐induced liver injury to occur. Therefore, immunotherapy is feasible for most chronic HBV or HCV cancer patients; however, in the treatment‐naïve patients, especially those with HCV infection, liver function and virological markers should be monitored carefully during ICI treatment.

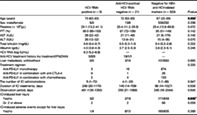

## Introduction

Immune checkpoint inhibitor (ICI) treatment shows antitumor effects through the suppression of immune inhibitory pathways targeting the programmed cell death protein‐1 (PD‐1)/programmed death‐ligand‐1 (PD‐L1) or the cytotoxic lymphocytes antigen proteins (CTLA‐4).^1^ ICI monotherapy and combination therapy with another ICI, cytotoxic agents, and/or molecular target agents have improved survival benefit and disease response in a wide range of malignancies from solid tumors^2^, ^3^, ^4^, ^5^, ^6^ to lymphoma.^7^ Adverse events due to ICIs are caused by the activation of the immune system and occur in many organ systems, causing pulmonary, musculoskeletal, gastrointestinal, ocular, endocrine, cardiovascular, and dermatologic disorders, which are called immune‐related adverse events (irAEs).^8^ In some cases, irAEs require not only temporary or permanent interruption of ICI treatment but also the administration of immunosuppressants, such as corticosteroids, mycophenolate mofetil, or infliximab, depending on their severity.^9^, ^10^

Hepatitis B virus (HBV) or hepatitis C virus (HCV) infection is a major health problem worldwide. The World Health Organization estimates that 257 million people have chronic HBV infection and 71 million people have chronic HCV infection.^11^ The rate of past HBV infection, chronic HBV infection, or chronic HCV infection in newly diagnosed cancer patients has been reported to be 6.5, 0.6, or 2.4%, respectively.^12^ Currently, large numbers of HBV‐ or HCV‐infected patients receive ICI treatment. In chronic hepatitis B or hepatitis C patients, the immune response is impaired as a result of the enhancement of PD‐1, PD‐L1, or CTLA‐4. Blocking PD‐1, PD‐L1, or CTLA‐4 by immunotherapy could clear virus‐infected hepatocytes by restoring the immune response against the viral antigen, but may also damage the liver.^13^ Liver injury has been previously reported to accompany virus reactivation^14^, ^15^, ^16^ or the decline of serum virus titer^16^, ^17^ during ICI treatment and requires treatment interruption, depending on the grade of severity. Hence, patients with chronic hepatitis B and hepatitis C are at considerable risk of developing liver injury during ICI treatment.

Increasing indications of ICI treatment necessitate assessing liver injury risk in HBV‐ or HCV‐infected cancer patients. A few investigators have previously analyzed the safety of ICI treatment in advanced cancer patients with HBV or HCV infection.^18^, ^19^, ^20^, ^21^ However, detailed recommendations or preventive measures required against liver injury during ICI treatment in HBV‐ or HCV‐infected patients remain unclear. We analyzed the frequency and severity of ICI‐induced liver injury in HBV‐ or HCV‐infected cancer patients to better address these points.

## Methods

### Study population

We retrospectively collected clinical data of patients with advanced malignancies who were treated with ICIs at the Osaka International Cancer Institute, Osaka, Japan, between November 2014 and September 2020. The administrated ICIs were anti‐PD‐L1 agents (atezolizumab, avelumab, or durvalmab), anti‐PD‐1 agents (nivolumab or pembrolizumab), or an anti‐CTLA‐4 agent (ipilimumab). Treatment regimens were ICI monotherapy or ICI combination therapy with another ICI, cytotoxic agents (paclitaxel [PTX], nab‐PTX, etoposide, cisplatin, carboplatin [CBDCA]), pemetrexed, 5‐fluorouracil, and/or molecular target agents (bevacizumab, axitinib). The present study was approved by the Institutional Review Board for Clinical Research at Osaka International Cancer Institute (approval number 21057).

### Assessment of ICI‐induced adverse events

The diagnosis of ICI‐induced adverse events was made by three physicians: one was the attending doctor of the patient, and the other two were the authors (Tasuku Nakabori and Yutaro Abe). The severity of ICI‐induced adverse events was retrospectively graded according to the National Cancer Institute Common Terminology Criteria for Adverse Events (CTCAE; version 5.0). ICI‐induced liver injury was diagnosed by excluding other causes of liver disease using medical interviews, blood tests including immunoglobulin G, immunoglobulin M, anti‐nuclear antibody, and anti‐mitochondrial antibody, and serological tests for hepatitis A, B, C, and E, and herpes simplex virus, and cytomegalovirus, or imaging modalities, such as ultrasonography, contrast‐enhanced computed tomography, or magnetic resonance imaging. The severity of ICI‐induced liver injury was defined as the severest CTCAE grade of alanine aminotransferase (ALT) or total bilirubin. Briefly, grade 1: ALT > upper limit of normal (ULN)—3.0 × ULN if baseline was normal, 1.5–3.0 × baseline if baseline was abnormal and/or total bilirubin > ULN—1.5 × ULN if baseline was normal, >1.0–1.5 × baseline if baseline was abnormal; grade 2: ALT > 3.0–5.0 × ULN if baseline was normal, >3.0–5.0 × baseline if baseline was abnormal and/or total bilirubin > 1.5–3.0 × ULN if baseline was normal, >1.5–3.0 × baseline if baseline was abnormal; grade 3: ALT > 5.0–20.0 × ULN if baseline was normal, >5.0–20.0 × baseline if baseline was abnormal and/or total bilirubin >3.0–10.0 × ULN if baseline was normal, >3.0–10.0 × baseline if baseline was abnormal; grade 4: ALT > 20.0 × ULN if baseline was normal, >20.0 × baseline if baseline was abnormal and/or total bilirubin > 10 × ULN if baseline was normal, >10 × baseline if baseline was abnormal; and grade 5: death. As for ICI‐induced adverse events other than liver injury, we analyzed events for grade ≥2, which are clinically significant and may require interruption of ICI treatment and initiation of immunosuppressants.^10^

### Measurement of virological markers

Serum HBV DNA and HCV RNA levels were measured using the COBAS TaqMan Test (Roche Diagnostics, Switzerland). The lower limit of detection of HBV DNA and HCV RNA was 1.3 and 1.2 log IU/mL, respectively. HBsAg, anti‐HBs, anti‐HBc, and anti‐HCV were measured using a chemiluminescent immunoassay system (CLIA System, Abbott Laboratories, North Chicago, IL, USA). The lower limit of detection of HBsAg was 0.05 IU/mL.

### Definition of HBV reactivation

In HBV‐related marker‐positive patients, serum HBV DNA was regularly monitored according to the Japan Society of Hepatology Guidelines for the Management of Hepatitis B Virus Infection.^22^ HBV reactivation was defined as the reappearance of serum HBV DNA higher than 1.3 log IU/mL from undetectable or below the lower limit of detection level of HBV DNA,^23^ or greater than a 10‐fold increase in HBV DNA from the baseline.^24^

### Statistical analysis

Continuous variables were expressed as median (range) and compared using the Mann–Whitney *U* test or Wilcoxon signed‐rank test, as appropriate. Kruskal–Wallis test and Bonferroni correction post hoc analysis were used for multiple comparisons. Categorical variables were expressed as numbers and compared using Pearson's chi‐square test or Fisher's exact test, as appropriate. A value of *P* < 0.05 was considered statistically significant. All statistical analyses were performed with SPSS version 20 (IBM Corp., Armonk, NY, USA).

## Results

### ICI‐induced liver injury in HBV patients and case presentation

In the present study, 266 patients with persistent or past HBV infection and 820 patients negative for HBV‐ and HCV‐related markers were identified. Table 1 and Tables [Link], [Link], Supporting information summarize the clinical characteristics before the ICI treatment and ICI‐induced adverse events. Baseline characteristics among HBsAg‐positive, anti‐HBc‐positive/anti‐HBs‐positive, anti‐HBc‐positive/anti‐HBs‐negative, and negative for HBV‐ and HCV‐related markers groups were compared. There were no significant differences in sex ratio, prothrombin time (PT), aspartate transaminase (AST), ALT, total bilirubin concentration, serum albumin level, distribution of cancer type of primary diagnosis, or existence of liver metastasis among the four groups. The ages of the participants were different among the four groups (*P* < 0.001); patients in the negative for HBV‐ and HCV‐related markers group tended to be younger than those in the other groups. Platelet count was also different among the four groups (*P* = 0.020).

**Table 1:** Baseline characteristics and immune checkpoint inhibitor‐induced adverse events in cancer patients with persistent or past hepatitis B virus (HBV) infection according to HBV‐related marker status

	HBsAg‐positive (*n* = 8)	Anti‐HBc‐positive/anti‐HBs‐positive (*n* = 190)	Anti‐HBc‐positive/anti‐HBs‐negative (*n* = 68)	Negative for HBV‐ and HCV‐related markers (*n* = 820)	*P*‐value
Age (years)	73 (50–83)	70 (48–90)	72 (47–85)	67 (22–89)	**<0.001** ^†^
Sex: male/female	7/1	140/50	47/21	538/282	0.106
Platelets (×10^4^/μL)	19.2 (17.4–21.6)	24.7 (9.3–64.8)	23.8 (10.2–67.9)	25.4 (13.9–69.8)	**0.020** ^‡^
PT^§^ (%)	94.0 (74–112)	97 (63–127)	92 (70–125)	95 (51–149)	0.129
AST (IU/L)	26 (17–39)	20 (10–63)	20 (10–123)	21 (8–175)	0.117
ALT (IU/L)	15.5 (8–26)	15 (5–83)	14 (4–70)	15 (4–95)	0.495
Total bilirubin (mg/dL)	0.6 (0.3–1.0)	0.5 (0.1–1.3)	0.4 (0.2–1.2)	0.4 (0.2–2.8)	0.416
Albumin, g/dl	4.0 (3.4–4.2)	3.8 (2.2–4.7)	3.8 (2.4–4.7)	3.8 (2.3–5.1)	0.428
HBV DNA (log IU/mL)	<1.3 (n.d.–4.6)	—	—	—	
HBsAg (IU/mL)	67.7 (0.21–250<)	—	—	—	
HBeAg, positive/negative	0/8	—	—	—	
Anti‐HBV treatment (naïve/NA)	4/4	—	—	—	
Liver metastasis, with/without	1/7	40/150	16/52	137/683	0.281
Treatment regimen					0.155
Anti‐PD‐(L)1 monotherapy	7	163	62	674	
Anti‐PD‐(L)1 in combination with anti‐CTLA‐4	1	7	0	26	
Anti‐PD‐(L)1 in combination with chemotherapy	0	20	6	120	
The number of ICI administrations	4 (1–32)	7 (1–57)	8 (1–56)	5 (1–88)	0.121
Duration of ICI treatment (days)	46 (14–476)	108 (14–1521)	132 (14–1253)	98 (14–1527)	0.185
Observation period (days)	228 (36–1115)	226 (17–1579)	394 (20–1702)	245 (16–2044)	0.233
ICI‐induced liver injury					
Yes/no	1/7	41/149	13/55	171/649	0.914
Gr. 2 or above	1	12	2	66	0.397
ICI‐induced adverse events except for liver injury					
Yes/no	1/7	48/142	20/48	185/635	0.389

^†^ Post hoc analysis showed that the patients in the negative for HBV‐ and HCV‐related markers group were younger than the patients in the anti‐HBc‐positive/anti‐HBs‐positive group and anti‐HBc‐positive/anti‐HBs‐negative group (*P* < 0.001, *P* < 0.001, respectively).

^‡^ Post hoc analysis showed that platelet count of the HBsAg‐positive group was lower than that of the anti‐HBc‐positive/anti‐HBs‐positive group, anti‐HBc‐positive/anti‐HBs‐negative, and negative for HBV‐ and HCV‐related markers group (*P* = 0.008, *P* = 0.013, *P* = 0.009, respectively).

^§^ Fifty two patients who were taking anticoagulants were excluded.

Continuous variables are shown as median (range). Bold numbers indicate the *P*‐value <0.05. The details about ICI‐induced adverse events except for liver injury are shown in Table S3.

—, none; ALT, alanine aminotransferase; AST, aspartate transaminase; HCV, hepatitis C virus; NA, nucleos(*t*)ides analog; n.d., not detected; PD, programmed cell death; PT, prothrombin time.

**Figure 1:**
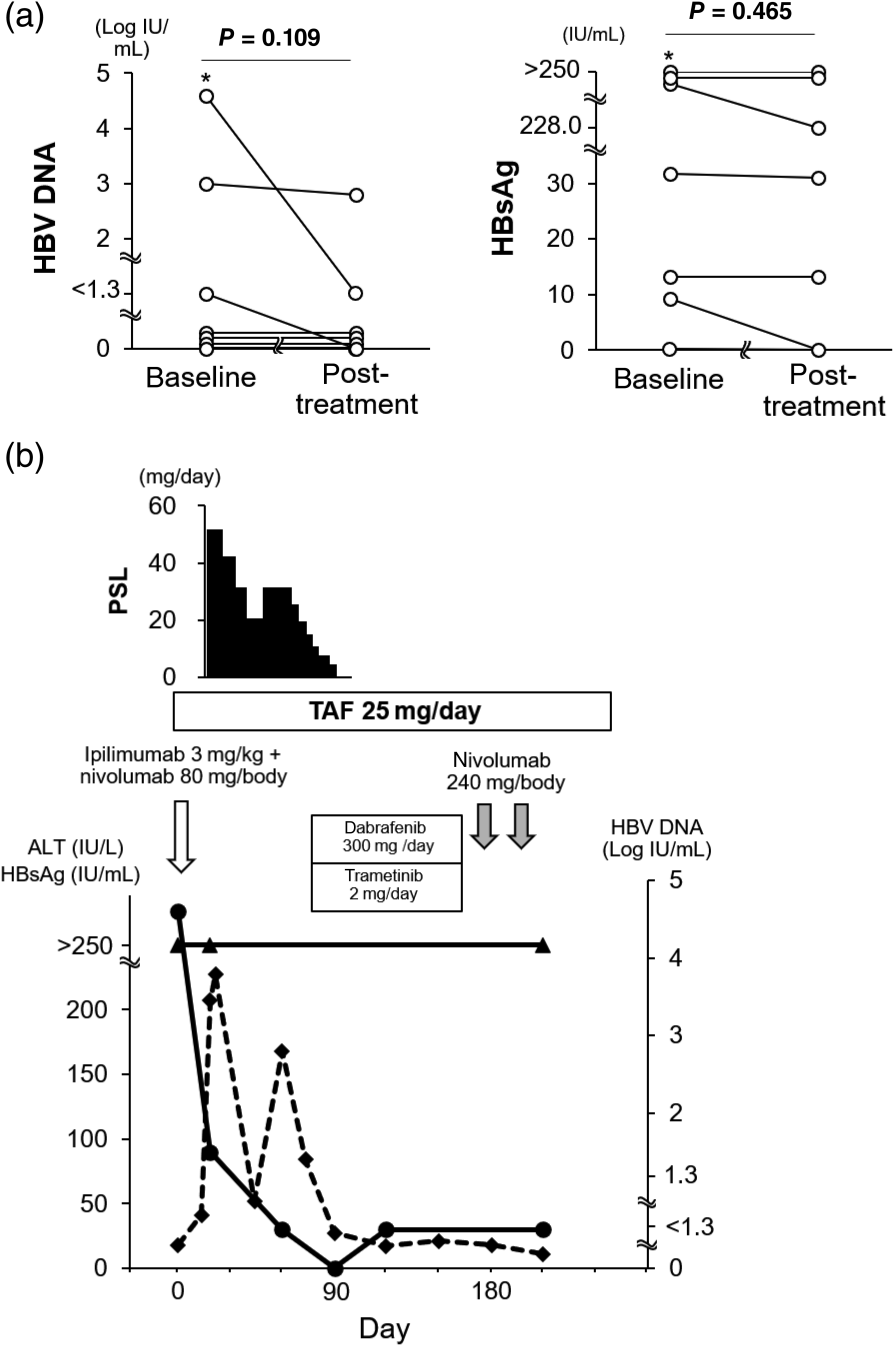
(a) Changes in serum hepatitis B virus (HBV) DNA and HBsAg levels during immune checkpoint inhibitor (ICI) treatment. Baseline: before or in the first course of ICI treatment. Post‐treatment: after the termination of ICI treatment. Asterisk (*) is the presented case in the results section for whom nucleotide analogs were initiated concurrently with ICI treatment. One patient who did not have the post‐treatment datum because of poor prognosis was excluded. (b) Case presentation in a chronic hepatitis B patient whose serum HBV DNA levels declined after the first administration of ipilimumab and nivolumab accompanied by grade 3 liver injury. Day 1 was defined as the day of the first administration of ipilimumab and nivolumab. (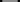), HBV DNA; (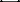), HBsAg; (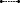), alanine aminotransferase (ALT). PSL, prednisolone; TAF, tenofovir alafenamide.

ICI treatment‐related factors and ICI‐induced adverse events were further compared. There were no differences in ICI regimen, the number of ICI administrations, duration of ICI treatment, and observation period among the four groups. There were no significant differences in frequency of any grade or ICI‐induced liver injury grade ≥2 or ICI‐induced adverse events other than liver injury. Of the patients with ICI‐induced liver injury, peak ALT levels and the time point of peak ALT levels were not different (Table S2). There was no HBV reactivation among 15 patients with ICI‐induced liver injury grade ≥2, although the serum HBV DNA level was missing from one patient. As for virus‐related factors in the HBsAg‐positive group, neither serum HBV DNA nor HBsAg level changed significantly during ICI treatment (Fig. 1a). There was one patient for whom antiviral treatment was initiated concomitantly with ICI treatment and whose serum HBV DNA level declined with grade 3 liver injury. The treatment‐naïve chronic hepatitis B patient was a 51‐year‐old woman with metastatic advanced malignant melanoma (Fig. 1b) and serum HBV DNA level of 4.6 log IU/mL. The patient started to take tenofovir alafenamide (TAF) concurrently with nivolumab and ipilimumab combination therapy. After the first administration of nivolumab and ipilimumab, serum ALT level increased, and serum HBV DNA level declined to 1.5 log IU/mL. Prednisolone (PSL) was initiated because serum ALT levels remained high. After that, serum ALT level temporarily improved, but increased again with an undetectable level of serum HBV DNA during the tapering period of the PSL dose. The dose of PSL was then increased, and the serum ALT level improved. The treatment regimen was changed to dabrafenib and trametinib combination therapy. Malignant melanoma progressed, and the treatment regimen was switched to nivolumab therapy. Liver injury did not recur under HBV suppression by oral administration of TAF during nivolumab monotherapy.

### ICI‐induced liver injury in HCV patients and case presentation

Of the patients screened, 35 were seropositive for anti‐HCV. Among them, nine patients without information on serum HCV RNA levels were excluded. Table 2 and Tables [Link], [Link] show the clinical characteristics before the ICI treatment and ICI‐induced adverse events in the remaining 26 patients. The baseline characteristics among the HCV RNA‐positive, anti‐HCV‐positive/HCV RNA‐negative, and negative for HBV‐ and HCV‐related markers groups were compared. There were no significant differences in sex ratio, platelet count, PT, AST, ALT, total bilirubin concentration, serum albumin level, distribution of cancer type of primary diagnosis, and existence of liver metastasis among the three groups. The ages of the patients were different among the three groups (*P* = 0.004); patients in the negative for HBV‐ and HCV‐related markers group tended to be younger than those in the other groups.

**Table 2:** Baseline characteristics and immune checkpoint inhibitor (ICI)‐induced adverse events in cancer patients positive for hepatitis C virus (HCV)‐related markers

	HCV RNA‐positive (*n* = 5)	Anti‐HCV‐positive/HCV RNA‐negative (*n* = 21)	Negative for HBV‐ and HCV‐related markers (*n* = 820)	*P*‐value
Age (years)	73 (63–83)	72 (53–83)	67 (22–89)	**0.004** ^†^
Sex: male/female	5/0	13/8	538/282	0.299
Platelets (× 10^4^/μL)	29.1 (13.2–41.1)	20.4 (11.2–39.5)	25.4 (13.9–69.8)	0.072
PT^‡^ (%)	98.0 (85–103)	87 (72–105)	95 (51–149)	0.142
AST (IU/L)	28 (22–42)	21 (11–40)	21 (8–175)	0.140
ALT (IU/L)	26 (12–32)	13 (4–31)	15 (4–95)	0.075
Total bilirubin (mg/dL)	0.6 (0.4–0.7)	0.5 (0.3–1.0)	0.4 (0.2–2.8)	0.332
Albumin (g/dL)	4.0 (3.8–4.3)	3.7 (2.5–5.0)	3.8 (2.3–5.1)	0.346
HCV RNA (log IU/mL)	6.3 (5.2–6.8)	—	—	
Anti‐HCV treatment history (no treatment/IFN/DAA)	5/0/0	19/1/1	—	
Liver metastasis, with/without	0/5	3/18	137/683	0.685
Treatment regimen				0.335
Anti‐PD‐(L)1 monotherapy	3	16	674	
Anti‐PD‐(L)1 in combination with anti‐CTLA‐4	0	1	26	
Anti‐PD‐(L)1 in combination with chemotherapy	2	4	120	
The number of ICI administrations	5 (1–72)	4 (1–32)	5 (1–88)	0.847
Duration of ICI treatments, days	248 (20–1170)	146 (14–709)	98 (14–1527)	0.556
Observation period, days	491 (138–1350)	259 (21–1058)	245 (16–2044)	0.540
ICI‐induced liver injury				
Yes/no	3/2	2/19	171/649	**0.045** ^§^
Gr. 2 or above	2	2	66	0.059
ICI‐induced adverse events except for liver injury				
Yes/no	1/4	8/13	185/635	0.389

^†^ Post hoc analysis showed that the patients in the negative for HBV‐ and HCV‐related markers group were younger than the patients in the anti‐HCV‐positive/HCV RNA‐negative group (*P* = 0.005).

^‡^ Thirty eight patients who are taking anticoagulants were excluded.

^§^ In post hoc analysis, there were no significant differences in any two groups' comparison.

Continuous variables are shown as median (range). Bold numbers indicate the *P*‐value <0.05. The details about ICI‐induced adverse events except for liver injury are shown in Table S6.

—, none; ALT, alanine aminotransferase; AST, aspartate transaminase; DAA, direct acting antiviral; IFN, interferon; PD, programmed cell death; PT, prothrombin time.

**Figure 2:**
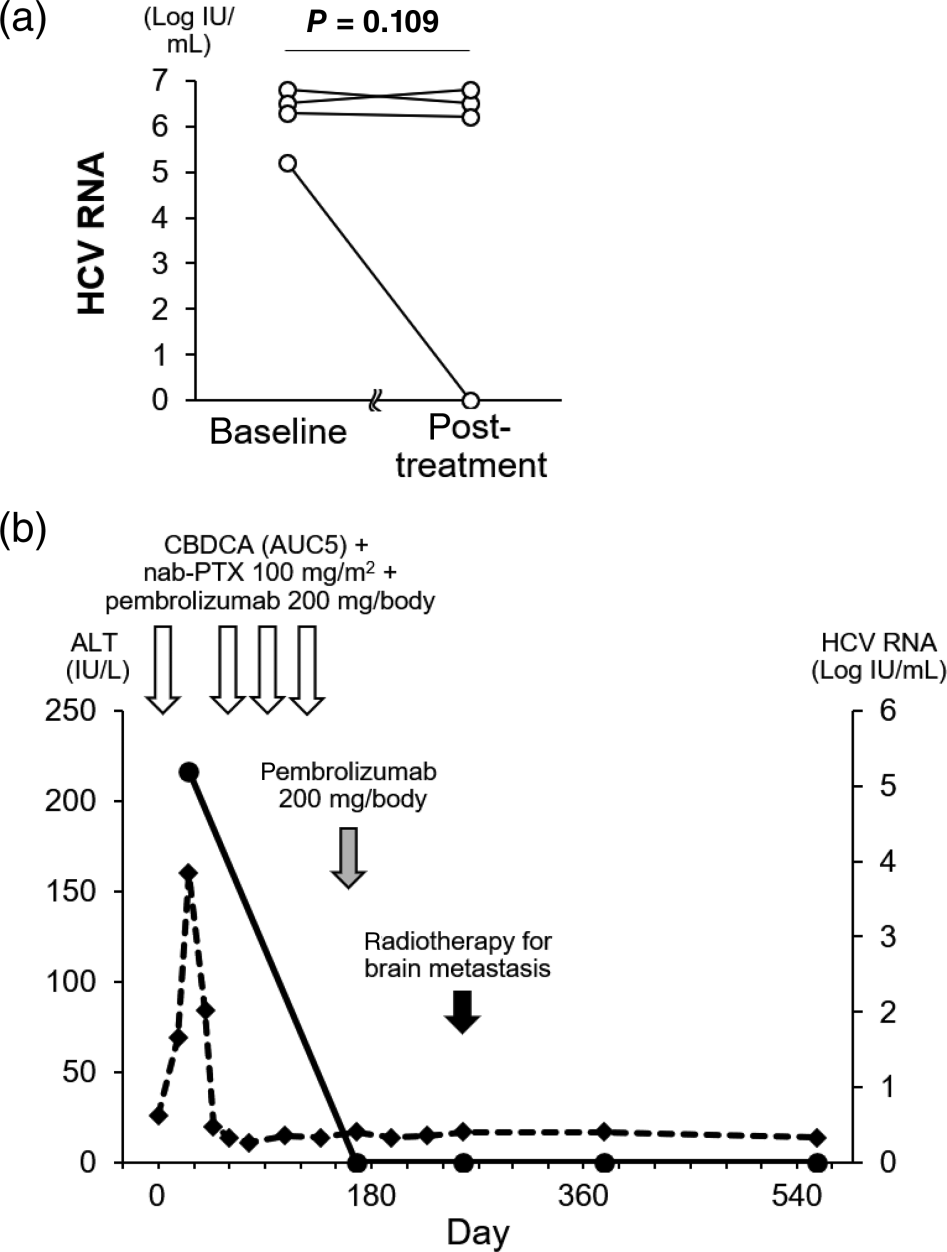
(a) Changes in serum hepatitis C virus (HCV) RNA level during ICI treatment. One patient who did not have post‐treatment data due to poor prognosis was excluded. (b) Case presentation in a chronic hepatitis C patient whose serum HCV RNA levels declined during the carboplatin (CBDCA), nab‐paclitaxel [PTX], and pembrolizumab therapy accompanied by grade 2 liver injury. Day 1 was defined as the day of the first administration of CBDCA, nab‐PTX, and pembrolizumab. (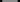), HCV RNA; (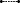), alanine aminotransferase (ALT).

ICI treatment‐related factors and ICI‐induced adverse events were further compared. There were no differences between the ICI regimen, the number of ICI administrations, duration of ICI treatment, and observation period. The frequency of any grade of ICI‐induced liver injury was different among the three groups (*P* = 0.045); it was observed in 3 of 5 HCV RNA‐positive patients (60.0%), compared to 2 of 21 anti‐HCV‐positive/HCV RNA‐negative patients (9.5%), and 171 of 820 patients with negative viral markers for HBV and HCV (20.9%). No significant difference was observed in the frequency of ICI‐induced liver injury grade ≥2. Of the patients with ICI‐induced liver injury, the time point of peak ALT level was not different; however, the peak ALT level was different among the three groups (Table S5). As for ICI‐induced adverse events other than liver injury, no significant difference was observed. The impact of the ICI treatment on HCV infection was evaluated in the HCV RNA‐positive group. ICI treatment did not change the serum HCV RNA level significantly (Fig. 2a). However, there was one patient whose serum HCV RNA level disappeared accompanied by grade 2 liver injury during ICI treatment. The patient was a 63‐year‐old man with metastatic advanced lung cancer and had no history of liver disease other than chronic hepatitis C (Fig. 2b). After the first administration of CBDCA, nab‐PTX, and pembrolizumab, the serum ALT level increased to 160 U/L at 5.2 log IU/mL of serum HCV RNA level. The ALT level declined shortly to within the normal range, and the patient resumed administration at the same dose. Liver injury relapse did not occur during the following administration of CBDCA, nab‐PTX, and pembrolizumab. Serum HCV RNA became undetectable 6 months after the liver injury at the first administration. Liver injury did not recur during pembrolizumab maintenance therapy, and serum HCV RNA level remained undetected for more than 1 year.

## Discussion

HBV and HCV are globally common infectious diseases. Patients infected with either virus are increasingly receiving ICI treatment along with expanding indication of ICIs for various malignancies. Immunotherapy in HBV‐ and HCV‐infected patients, whose immune responses are impaired, could induce liver injury due to clearance of virus‐infected hepatocytes by restoring the immune response against viral antigens. Therefore, the risk of exacerbation of hepatitis is a concern in patients with HBV or HCV, compared to those without it, during ICI treatment. To better understand this, we investigated the frequency and severity of liver injury in cancer patients with HBV or HCV infection who underwent ICI treatment.

In the comparison of clinical characteristics among the patients with persistent and past HBV infection and those who were negative for HBV‐ and HCV‐related markers, HBsAg‐positive patients had a lower platelet count than those with anti‐HBc‐positive/anti‐HBs‐positive, anti‐HBc‐positive/anti‐HBs‐negative, and negative for HBV‐ and HCV‐related markers. This may be because progressive chronic liver disease was more severe in patients with persistent HBV infection than in those with temporary HBV infection.^25^ As for the rate of any grade ICI‐induced liver injury, the occurrence rates were 12.5, 21.6, 19.1, and 20.9% in the HBsAg‐positive, anti‐HBc‐positive/anti‐HBs‐positive, anti‐HBc‐positive/anti‐HBs‐negative, and negative for HBV‐ and HCV‐related markers groups, respectively, with no significant differences among the groups. The frequency of liver injury in all groups was comparable to that in the general patients; previous studies have reported that 8.7–22.3% of patients develop liver injury due to ICI treatment.^3^, ^4^, ^5^ Our result also showed that, among the HBsAg‐positive patients, the levels of neither serum HBV DNA nor HBsAg changed significantly during ICI treatment. Furthermore, in our investigation, no HBV reactivation was seen in cases of liver injury of grade ≥2. These findings indicate that HBV infection may not have a clinically significant impact on liver function in most cases of cancer patients treated with ICIs.

With respect to HCV infection, a higher tendency of any grade ICI‐induced liver injury to occur was observed in the HCV RNA‐positive group, compared to the anti‐HCV‐positive/HCV RNA‐negative and negative for HBV‐ and HCV‐related markers groups, which suggests that liver inflammation may be facilitated by ICI treatment in patients with persistent HCV infection. On the other hand, no significant difference was observed in grade ≥2 ICI‐induced liver injury between the HCV RNA‐positive and ‐negative groups. Regarding virus dynamics, ICI treatment did not change serum HCV RNA levels. Taken together, HCV infection may not have a considerable influence on ICI treatment in most cancer patients.

Regarding the relationship between HCV infection and ICI‐induced liver injury, a tendency for higher peak ALT levels was observed in the anti‐HCV‐positive/HCV RNA‐negative groups because the two patients with ICI‐induced liver injury in this group had severe liver damage. In this regard, it cannot be suggested that ICI‐induced liver injury in the patients with previous HCV infection is more severe than in patients who are negative for HBV‐ and HCV‐related markers, because the number of patients with ICI‐induced liver injury in the anti‐HCV‐positive/HCV RNA‐negative group was quite small. In addition, the anti‐HCV‐positive/HCV RNA‐negative group may have included patients who were false positive for anti‐HCV. Further investigation is required on this point.

We encountered one HBV‐infected patient and one HCV‐infected patient who had liver injury accompanied by a decline of serum virus level during ICI treatment. Both patients were treatment‐naïve. The presented HBV‐infected patient initiated antiviral treatment concomitantly with ICI treatment. The patient showed bimodal ALT elevation, which was improved by oral PSL therapy. Thus, the decline of serum HBV DNA level may be due to antiviral therapy, and liver injury appeared to be immune‐related, caused by ICI treatment, though the involvement of clearance of HBV‐infected hepatocytes by ICI‐induced immune restoration against viral antigens could not be completely excluded. On the contrary, liver injury was more likely in the presented HCV‐infected patient due to ICI‐mediated elimination of virus‐infected hepatocytes by restoring the immune response against viral antigens than in the above‐mentioned HBV‐infected patient because HCV infection rarely resolves spontaneously once it becomes chronic.^26^ Nevertheless, HCV disappeared with substantial ALT increase after the administration of ICI. Serum HCV RNA was negative for more than 1 year, which was just like sustained virological response by DAAs (direct acting antivirals) or interferon treatment. Thus, these findings showed that liver injury accompanied by decline of serum HCV RNA level could occur in treatment‐naïve patients as described in the previous case reports.^27^, ^28^ In particular, as far as we know, this is the first report regarding a persistently HCV‐infected cancer patient in whom eradication of the virus was observed during ICI treatment.

Our study had several important limitations, including its retrospective nature, single‐center study, small sample size, and lack of some clinical data. Further investigation is required to validate our results. On the other hand, previous studies regarding the safety of ICI treatment in HBV‐ or HCV‐infected patients mainly included patients with limited types of malignancies, such as melanoma, lung cancer, and HCC, or were focused on ICI treatment alone.^18^, ^19^, ^20^, ^21^ ICI treatment is being increasingly indicated for malignancies and tends to be administered with a concurrent cytotoxic agent or a molecular target agent. This report could reflect real‐world outcomes because our study included a wide range of malignancies and ICI‐combined treatment with other traditional agents.

In conclusion, neither HBV nor HCV infection has a considerable impact on the safety of ICI treatment. Therefore, ICI treatment is feasible for most chronic HBV‐ or HCV‐infected cancer patients. On the other hand, in treatment‐naïve patients, especially those with HCV infection, liver function, and virological markers, should be carefully monitored during ICI treatment because virus‐related liver injury could be induced by ICI treatment.

## Supporting information


**Table S1.** Cancer type of primary diagnosis in patients with persistent or past HBV infection.

**Table S2.** Peak ALT level and its time point in persistent or past HBV‐infected patients with ICI‐induced liver injury.

**Table S3.** ICI‐induced adverse events except for liver injury in persistent or past HBV‐infected patients.

**Table S4.** Cancer type of primary diagnosis in patients positive for HCV‐related markers.

**Table S5.** Peak ALT level and its time point in patients positive for HCV‐related markers with ICI‐induced liver injury.

**Table S6.** ICI‐induced adverse events except for liver injury in cancer patients positive for HCV‐related markers.

## Data Availability

The data shown in the present study are available from the corresponding author, upon reasonable request.
